# Familywise error rate control for block response-adaptive randomization

**DOI:** 10.1177/09622802231167437

**Published:** 2023-04-06

**Authors:** Ekkehard Glimm, David S Robertson

**Affiliations:** 1 Novartis Pharma AG, Basel, Switzerland; 2 Medical Faculty, Institute of Biometry and Medical Informatics, University of Magdeburg, Magdeburg, Germany; 3 MRC Biostatistics Unit, University of Cambridge, Cambridge, UK

**Keywords:** Conditional invariance principle, multiple testing, power, type I error rate

## Abstract

Response-adaptive randomization allows the probabilities of allocating patients to treatments in a clinical trial to change based on the previously observed response data, in order to achieve different experimental goals. One concern over the use of such designs in practice, particularly from a regulatory viewpoint, is controlling the type I error rate. To address this, Robertson and Wason (Biometrics, 2019) proposed methodology that guarantees familywise error rate control for a large class of response-adaptive designs by re-weighting the usual z -test statistic. In this article, we propose an improvement of their method that is conceptually simpler, in the context where patients are allocated to the experimental treatment arms in a trial in blocks (i.e. groups) using response-adaptive randomization. We show the modified method guarantees that there will never be negative weights for the contribution of each block of data to the adjusted test statistics, and can also provide a substantial power advantage in practice.

## 1. Introduction

Randomized clinical trials are often designed in such a way that a decision about treatment efficacy is reached as quickly as possible and with a minimum number of patients exposed to inferior treatment options. Response-adaptive randomization (RAR) can help achieve such goals by an allocation process that makes randomization of a newly recruited patient dependent on responses to treatment from previous study participants. This can offer advantages in terms of the benefit for patients recruited into the study, increasing the willingness of patients to participate in the study, or the study’s power to detect treatment effects. Many different classes of RAR procedures have been proposed for various trial contexts, and a recent review of methodological and practical issues around the use of RAR in clinical trials can be found by Robertson et al.^1^ However, one major concern is about the potential for type I error rate inflation arising from such studies. This is particularly the case from a regulatory viewpoint, where control of the type I error rate is required for confirmatory studies.^2,3^

Robertson and Wason^4^ proposed a methodology that guarantees a type I error rate control for normally distributed outcomes by reweighting the usual z -statistic through iterative application of the conditional invariance (CIV) principle. For that purpose, they assume the existence of a hypothetical ‘auxiliary design’ in which the test statistic has a known null distribution. At interim analyses of the RAR trial (this may be after blocks/groups of patients or after every patient), the randomization ratios may be changed. The test statistic used to test for treatment efficacy, however, is calculated so that its null distribution matches the null distribution of the test statistic from the auxiliary design. Robertson and Wason^4^ show that this guarantees type I error control at level α if the test in the auxiliary design is a level α test. For multi-arm trials, the testing procedure controls the familywise error rate (FWER), which is defined as the maximum probability of at least one type I error under any configuration of true and false null hypotheses.

In this article, we present an improvement of their proposal based on the CIV principle. It is simpler in that it requires only modifying the final test statistic so that it has a known variance, and not a known mean. This restricts the method to trials where the patients are allocated to the experimental arms in blocks (i.e. groups) using RAR while the allocation to the control arm is fixed. On the other hand, by construction, the method guarantees that there will never be negative weights for the contribution of each block of data to the adjusted test statistic, and the modified method can also provide a substantial power advantage in practice.

The outline of the rest of the article is as follows. In Section 2, we describe the proposed testing procedure and its connection with existing approaches. A simulation study is presented in Section 3 to compare the testing strategies, and a case study is given in Section 4. We conclude with a discussion in Section 5.

## 2. Proposed testing procedure

Consider a trial with K≥1 experimental treatment arms and a common control arm. We assume that the allocation of patients to the control arm is fixed throughout the trial, so that there are a total of $n_{0}$ control observations. For the treatment arms, there is a ‘burn-in period’ which allocates b patients according to a predefined allocation scheme. After the end of this burn-in, patients are allocated in blocks to the treatment arms using a RAR scheme. In total, there are n patients allocated to the experimental treatments in the trial across B blocks.

For simplicity, we consider testing each elementary null hypothesis $H_{k}:\mu_{k}=\mu_{0}$ , where $\mu_{k}$ and $\mu_{0}$ are the expected responses from patients allocated to experimental treatment k and the control, respectively. Extensions to intersection hypotheses within a closed test procedure will be discussed in Section 2.2. Let $a_{i}$ denote the treatment allocation for the i -th patient on the experimental treatment arms, where $a_{i}=k$ if the patient i is allocated to experimental treatment k . We assume that each of the observations on the control arm follow a $N(\mu_{0},\sigma^{2})$ distribution, while $X_{i}\;|\;(a_{i}=k)∼N(\mu_{k},\sigma^{2})$ is the response obtained from patient i given that they are allocated to experimental treatment group k . The variance $\sigma^{2}$ is assumed known and, without loss of generality, we set $\sigma^{2}=1$ .

The standard z -test for $H_{k}$ uses the test statistic ${\bar{X}}_{k}-{\bar{X}}_{0}$ , where ${\bar{X}}_{0}$ and ${\bar{X}}_{k}$ are the mean response for patients on the control and treatment k , respectively. However, if RAR is used, the distribution of the z -test is affected and hence the type I error rate may no longer be controlled, with the FWER being highly inflated under certain types of RAR.

In order to tackle this issue, like in Robertson and Wason^4^ we introduce a hypothetical ‘auxiliary design,’ which can be thought of as one of the allowed randomization lists, chosen before the beginning of the trial. Unless there are reasons to choose otherwise, a default option would be to use fixed (equal) randomization to reflect the uncertainty before the trial begins over which of the treatment options will be superior. Since the allocations to the treatments are all fixed in advance in the auxiliary design, the standard z -test is a valid test that controls the type I error rate.

For the auxiliary design, let $b_{i}$ denote the treatment allocation for the i -th patient on the experimental treatment arms. We assume $Y_{i}\;|\;(b_{i}=k)∼N(\mu_{k},1)$ is the response of patient i , given that they were allocated to treatment group k in the auxiliary design. For i≤b (i.e. during the burn-in period), we simply set $a_{i}=b_{i}$ . Afterwards, the two designs diverge according to the RAR scheme used.

In this setup, the actual trial design (i.e. the realized allocations of patients to treatments using RAR) can be viewed as a series of data-dependent modifications of the auxiliary design, where we account for these modifications using the CIV principle. Robertson and Wason^4^ proposed a modified test statistic ${\tilde{T}}_{k}$ for testing the hypothesis $H_{k}$ . This test statistic is a difference of weighted means of the observations on the treatment arm k and the control arm, where the weights are calculated recursively based on the number of allocations to the experimental treatments and the control.

We now describe a simplification of the handling of the control group in this setup. The basic idea of the modified proposal is to only match the variances of the test statistics under the auxiliary and actual trial designs, and not matching the means. Due to this, the CIV principle may no longer be used as a justification for the algorithm, but a minor modification of it still applies.

Let ${\bar{X}}_{k}(n_{0,k})∼N(\mu_{k},\frac{1}{n_{0,k}})$ be the mean response in treatment group k after the burn-in period, where $n_{0,k}$ patients have been allocated to treatment k according to a pre-specified allocation scheme. For each subsequent block j of recruited patients ( j=1,…,B ), the randomization probability for treatment k may be changed in some way such that in block j , we have ${\tilde{n}}_{j,k}$ patients in treatment arm k instead of the pre-planned $n_{j,k}$ patients from the auxiliary design. We proceed in this fashion up to a final block B . In every block, we assume that at least one patient will be randomized to each treatment k . Finally, we let $m_{j,k}=n_{j,k}+\ldots +n_{B,k}$ denote the sum of the sample sizes of treatment group k in blocks j,…,B of the auxiliary design. For notational simplicity, we define $m_{0,k}=n_{k}$ , that is, $n_{k}$ is the total number of patients allocated to treatment k in the auxiliary design.

The following formulae define the resulting summary statistics and their (conditional) distributions:

**Block 0**: This is the burn-in period. After this block, we define $U_{0}=\frac{n_{0,k}}{n_{k}}{\bar{X}}_{k}(n_{0,k})∼N(\frac{n_{0,k}}{n_{k}}\mu_{k},\frac{n_{0,k}}{n_{k}^{2}})$ and set $w_{0}=n_{k}$ .

**Block 1**: We define the following test statistics: Weighted data from block 1 of the auxiliary design:

$$U_{1}=\frac{n_{1,k}}{w_{0}}{\bar{Y}}_{k}(n_{1,k})∼N\left(\frac{n_{1,k}}{w_{0}}\mu_{k},\frac{n_{1,k}}{w_{0}^{2}}\right)$$

Weighted data from block 1 of the actual design:

$${\tilde{U}}_{1}=\frac{{\tilde{n}}_{1,k}}{w_{1}}{\bar{X}}_{k}({\tilde{n}}_{1,k})∼N\left(\frac{{\tilde{n}}_{1,k}}{w_{1}}\mu_{k},\frac{{\tilde{n}}_{1,k}}{w_{1}^{2}}\right)$$

Weighted data from blocks 2,…,B of the auxiliary design:

$$U_{\left(2\right)}=\frac{m_{2,k}}{w_{1}}{\bar{Y}}_{k}(m_{2,k})∼N\left(\frac{m_{2,k}}{w_{1}}\mu_{k},\frac{m_{2,k}}{w_{1}^{2}}\right)$$

Here, ${\bar{Y}}_{k}(n_{1,k})$ is the mean response of patients in block 1 who are allocated to treatment k in the auxiliary design, ${\bar{X}}_{k}({\tilde{n}}_{1,k})$ is the mean response of patients in block 1 who were allocated to treatment k in the actual design, and ${\bar{Y}}_{k}(m_{2,k})$ is the mean response of patients in all blocks after block 1 who are allocated to treatment k in the auxiliary design. Since ${\tilde{n}}_{1,k}$ is a function of the entire data from the patients in block 0, the given distribution of ${\tilde{U}}_{1}$ is conditional on the block 0 data.

We now form the overall test statistics for blocks 1,2,…,B in the trial, $U_{\left(1\right)}=U_{1}+U_{\left(2\right)}$ and ${\tilde{U}}_{\left(1\right)}={\tilde{U}}_{1}+U_{\left(2\right)}$ , using the block 1 data from the auxiliary and actual designs, respectively. We then choose the weight $w_{1}$ so that the conditional variances of $U_{\left(1\right)}$ and ${\tilde{U}}_{\left(1\right)}$ are matched. Hence, $w_{1}=w_{0}\cdot \sqrt{\frac{{\tilde{n}}_{1,k}+m_{2,k}}{n_{1,k}+m_{2,k}}},$ leading to the conditional distribution given the block 0 data as ${\tilde{U}}_{\left(1\right)}∼N(\frac{{\tilde{n}}_{1,k}+m_{2,k}}{w_{1}}\mu_{k},\frac{m_{1,k}}{w_{0}^{2}}).$

**Block** j∈{2,…,B−1} : After every block, the randomization probabilities may be changed. For block j we define the following test statistics: Weighted data from block j of the auxiliary design:

$$U_{j}=\frac{n_{j,k}}{w_{j-1}}{\bar{Y}}_{k}(n_{j,k})∼N\left(\frac{n_{j,k}}{w_{j-1}}\mu_{k},\frac{n_{j,k}}{w_{j-1}^{2}}\right)$$

Weighted data from block j of the actual design:

$${\tilde{U}}_{j}=\frac{{\tilde{n}}_{j,k}}{w_{j}}{\bar{X}}_{k}({\tilde{n}}_{j,k})∼N\left(\frac{{\tilde{n}}_{j,k}}{w_{j}}\mu_{k},\frac{{\tilde{n}}_{j,k}}{w_{j}^{2}}\right)$$

Weighted data from blocks j+1,…,B of the auxiliary design:

$$U_{\left(\;j+1\right)}=\frac{m_{j+1,k}}{w_{j}}{\bar{Y}}_{k}(m_{j+1,k})∼N\left(\frac{m_{j+1,k}}{w_{j}}\mu_{k},\frac{m_{j+1,k}}{w_{j}^{2}}\right)$$

Here ${\bar{Y}}_{k}(n_{j,k})$ is the mean response of patients in block j on treatment k in the auxiliary design, ${\bar{X}}_{k}({\tilde{n}}_{j,k})$ is the mean response of patients in block j on treatment k in the actual design, and ${\bar{Y}}_{k}(m_{j+1,k})$ is the mean response of patients in blocks j+1,…,B on treatment k in the auxiliary design. Since ${\tilde{n}}_{j,k}$ is a function of the entire data from the patients in blocks 0,1,…,j−1 , the given distribution of ${\tilde{U}}_{j}$ is conditional on these data.

We form the overall test statistics for blocks j,j+1,…,B in the trial, $U_{\left(\;j\right)}=U_{j}+U_{\left(\;j+1\right)}$ and ${\tilde{U}}_{\left(\;j\right)}={\tilde{U}}_{j}+U_{\left(\;j+1\right)}$ , using the block j data from the auxiliary and actual designs, respectively. We choose the weight $w_{j}$ so that the conditional variances of $U_{\left(\;j\right)}$ and ${\tilde{U}}_{\left(\;j\right)}$ are matched. Hence

$$w_{j}=w_{j-1}\cdot \sqrt{\frac{{\tilde{n}}_{j,k}+m_{j+1,k}}{n_{j,k}+m_{j+1,k}}}$$

Consequentially, the conditional distribution of ${\tilde{U}}_{\left(\;j\right)}$ given the data from blocks 0,…,j−1 is

$${\tilde{U}}_{\left(\;j\right)}∼N\left(\frac{{\tilde{n}}_{j,k}+m_{j+1,k}}{w_{j}}\mu_{k},\frac{m_{j,k}}{w_{j-1}^{2}}\right)$$

**Final block** B : We define

$$\begin{array}{rl} & U_{B}=\frac{n_{B,k}}{w_{B-1}}{\bar{Y}}_{k}(n_{B,k})∼N\left(\frac{n_{B,k}}{w_{B-1}}\mu_{k},\frac{n_{B,k}}{w_{B-1}^{2}}\right)\\ & {\tilde{U}}_{B}=\frac{{\tilde{n}}_{B,k}}{w_{B}}{\bar{X}}_{k}({\tilde{n}}_{B,k})∼N\left(\frac{{\tilde{n}}_{B,k}}{w_{B}}\mu_{k},\frac{{\tilde{n}}_{B,k}}{w_{B}^{2}}\right) \end{array}$$

Since no additional block follows, we match the conditional variances of $U_{B}$ and ${\tilde{U}}_{B}$ , so that $w_{B}=w_{B-1}\cdot \sqrt{\frac{{\tilde{n}}_{B,k}}{n_{B,k}}}$ and hence ${\tilde{U}}_{\left(B\right)}={\tilde{U}}_{B}∼N(\frac{{\tilde{n}}_{B,k}}{w_{B}}\mu_{k},\frac{n_{B,k}}{w_{B-1}^{2}}).$

**Final test statistic**: At the end of this procedure, we obtain the test statistic $\tilde{U}=U_{0}+\sum_{j=1}^{B}{\tilde{U}}_{j}$ . The corresponding statistic from the auxiliary design is $U=\sum_{j=0}^{B}U_{j}$ . By construction, both U and $\tilde{U}$ have variance $\text{var}(U)=\frac{1}{n_{k}}$ . However, while $E(U)=\mu_{k}$ , $E(\tilde{U})$ cannot be easily derived due to the response-adaptive modifications.

Since we did not modify the randomization to the control treatment, we have a consistent estimate of $\mu_{0}$ from ${\bar{X}}_{0}(n_{0})∼N(\mu_{0},\frac{1}{n_{0}})$ , where $n_{0}$ is the total number of patients on the control and ${\bar{X}}_{0}(n_{0})$ is the mean response in the control group across all blocks. If we had known $\mu_{0}$ at the time of doing the blockwise-RAR, we could simply have subtracted $\mu_{0}$ from every observation $X_{i}$ and run the algorithm in the described way on $X_{i}-\mu_{0}$ instead of $X_{i}$ . Since $E(X_{i}\;|\;a_{i}=k)=\mu_{k}=\mu_{0}$ under $H_{k}$ , this would have led to $E({\tilde{U}}_{j})=0$ for j=0,1,…,B and removed the need to match the weights on the means as well. As we have a record of all weights $w_{1},\ldots ,w_{B}$ , we can do this ‘post-hoc’ using ${\bar{X}}_{0}(n_{0})$ as a consistent estimator of $\mu_{0}$ :

$$\begin{array}{rl} {\tilde{U}}^{*} & =U_{0}-\frac{n_{0,k}}{n_{k}}{\bar{X}}_{0}(n_{0})+\sum_{j=1}^{B}\left[{\tilde{U}}_{j}-\frac{{\tilde{n}}_{j,k}}{w_{j}}{\bar{X}}_{0}(n_{0})\right]\\ & =\tilde{U}-\sum_{j=0}^{B}v_{j}{\bar{X}}_{0}(n_{0}) \end{array}$$

where $v_{j}=\frac{{\tilde{n}}_{j,k}}{w_{j}}$ for j=0,1,…,B and ${\tilde{n}}_{0,k}=n_{0,k}$ . The final test statistic is then $\tilde{T}=\frac{{\tilde{U}}^{*}}{\text{sd}({\tilde{U}}^{*})},$ where $\text{sd}({\tilde{U}}^{*})=\sqrt{\frac{1}{n_{k}}+(\sum_{j=0}^{B}v_{j}{)}^{2}\frac{1}{n_{0}}}$ . $\tilde{T}$ is distributed as N(0,1) under $H_{k}$ if $\sigma^{2}=1$ is known.

If $\sigma^{2}$ is not known, and hence must be estimated from the data, $\tilde{T}/\hat{\sigma }$ is asymptotically normally distributed.

Regarding the estimation of $\sigma^{2}$ , the suggested approach is no different from other group-sequential or adaptive designs. The most natural choice would be to use usual pooled estimators ${\hat{\sigma }}_{j}^{2}=\frac{1}{\sum_{k=0}^{K}{\tilde{n}}_{j,k}-K-1}\sum_{k=0}^{K}\sum_{i=1}^{{\tilde{n}}_{j,k}}(x_{ijk}-{\bar{x}}_{jk}{)}^{2}$ per block j from the observed individual responses $x_{ijk}$ and then combine these in some appropriate way (e.g. by summing them up with equal weights $\frac{1}{\sqrt{j+1}}$ after every block j ). Since the estimate of mean and variance are stochastically independent in this setting, these per-block estimates are all consistent estimates of $\sigma^{2}$ .

**Early stopping:** The approach can be modified to allow early stopping for efficacy if the number of control patients is fixed per block. In that case, we can setup the approach similar to a group-sequential trial with α -spending. Rather than calculating $\tilde{T}$ only once at the end of the trial after block B , we would allow an interim analysis after block S<B . The corresponding test statistic ${\tilde{T}}_{\left(S\right)}$ is calculated by treating S as the last block (i.e. setting B=S in the above algorithm for $\tilde{T}$ ) and using ${\bar{X}}_{0}(n_{S,0})$ instead of ${\bar{X}}_{0}(n_{0})$ , where $n_{S,0}$ is the total number of patients allocated to the control in blocks 0,1,…,S and $\bar{X}(n_{S,0})$ is the mean response of these patients. Since the number of control observations in blocks 0,1,…,B are fixed in advance, ${\bar{X}}_{0}(n_{S,0})∼N(\mu_{0},\frac{1}{n_{S,0}})$ such that ${\tilde{T}}_{\left(S\right)}∼N(0,1)$ still holds.

If there is a sequence $\alpha_{1},\ldots ,\alpha_{B}$ with $\sum_{j=1}^{B}\alpha_{j}=\alpha$ , then rejecting $H_{k}$ when ${\tilde{T}}_{\left(S\right)}\geq \Phi^{-1}(1-\alpha_{S})$ controls the type I error rate for treatment k by the Bonferroni inequality. In ordinary group-sequential designs, the correlation between test statistics from different stages is known (Jennison and Turnbull^5^), allowing an improvement over Bonferroni (i.e. assigning a sequence of test levels $\alpha_{1},\ldots ,\alpha_{B}$ with a sum larger than α , but still preserving the type I error of α ). Unfortunately, this improvement is not available here since the correlation between ${\tilde{T}}_{\left(S\right)}$ and $\tilde{T}$ depends on $v_{S+1},\ldots ,v_{B}$ which are not available at the time of the interim analysis. The correlation *could* be calculated if after the interim analysis, the auxiliary design would be strictly followed for blocks S+1,…,B . However, this would defeat the purpose of RAR.

Note that a test of ${\tilde{T}}_{\left(S\right)}$ at level α at a random time S would only control the type I error rate if S was stochastically independent of ${\tilde{T}}_{\left(1\right)},\ldots ,{\tilde{T}}_{\left(S-1\right)}$ under $H_{k}$ . Clearly, calculating ${\tilde{T}}_{\left(j\right)}$ after every block and then deciding when to test based on the observed value could lead to an inflation of the type I error rate.

### 2.1. Connection with existing approaches

There is a close connection between the proposal of Robertson and Wason^4^ (and hence our modified proposal) and more traditional adaptive designs. Assume that we want to test the one-sample hypothesis $H_{0}:\mu =\mu_{0}$ adaptively. Before the start of the study, we plan a first interim analysis after $m_{0}$ patients and a weight $w_{0}\leq 1$ for this first step. At the interim, we calculate the test statistic $T_{0}=\sqrt{m_{0}}(\bar{X}(m_{0})-\mu_{0})$ which is N(0,1) under $H_{0}$ . Subsequently, we pick a new sample size ${\tilde{m}}_{1}$ for the next stage (up to the next interim) and a weight $w_{1}$ for this stage. The weight and ${\tilde{m}}_{1}$ may both depend on the data from stage 0, but the restriction $w_{0}^{2}+w_{1}^{2}\leq 1$ must be obeyed. At the end of the first stage, we calculate $T_{1}=\sqrt{{\tilde{m}}_{1}}(\bar{X}({\tilde{m}}_{1})-\mu_{0})$ given the stage 0 data and $T_{\left(1\right)}=w_{0}T_{0}+\sqrt{(1-w_{0}{)}^{2}}T_{1}$ , which are both N(0,1) under $H_{0}$ .

At the next stage (stage 2), we pick a new sample size ${\tilde{m}}_{2}$ and weight $w_{2}$ for the following stage, with $w_{0}^{2}+w_{1}^{2}+w_{2}^{2}\leq 1$ . We combine the test statistics $T_{2}=\sqrt{{\tilde{m}}_{2}}(\bar{X}({\tilde{m}}_{2})-\mu_{0})$ and $T_{\left(1\right)}$ using $w_{1}$ , that is, by forming a test statistic $T_{\left(2\right)}=w_{1}T_{\left(1\right)}+\sqrt{(1-w_{1}{)}^{2}}T_{2}$ . We continue in this fashion until at some point we decide to call the final analysis B . Then, at the penultimate analysis (the last interim before this final one), we spend the rest of the weights such that $\sum_{j=1}^{B}w_{j}^{2}=1$ . Hence, the last weight $w_{B}=\sqrt{1-\sum_{j=1}^{B-1}w_{j}^{2}}$ is fixed by design. The weights $w_{j}$ are allowed to depend on all data up to interim analysis j−1 , so the weights from later stages can be iterated updates of previous weights. This approach was described by Brannath et al.^6^ The proposals described in this article and by Robertson and Wason^4^ can be viewed as an application of this general procedure, with a special way of calculating the weights and a ‘time horizon’ (in terms of the total number of patients) which is given from the start. In addition, the method described in this article does not combine standardized stagewise test statistics directly, but rather corrects for fluctuations in the expected values by adjustment at the end of the trial.

### 2.2. Application within a closed test procedure

The approach described above for controlling the type I error rate for a single hypothesis $H_{k}$ generalizes to controlling the FWER by applying a closed test procedure (CTP). In the CTP (Marcus et al.^7^), $H_{k}$ , k=1,…,K is rejected if and only if all $H_{K}$ , K⊆{1,…,K} with k∈K are rejected at level α where $H_{K}=\cap_{k\in K}H_{k}$ . In order to test $H_{K}:\mu_{k}=\mu_{0}\;\forall k\in K$ , several approaches can be considered. For example:

- All observations of the experimental treatment arms in K are pooled and treated as a single treatment arm to test against the control treatment with the approach described in Section 2. This is called ‘closed z -test’ in the simulations below. This approach will lead to low power unless all treatments in K are truly effective. Otherwise, the test statistic for the treatments in K will be ‘diluted’ towards the null by the contribution of the treatments in K that are truly null.
- Assume that the approach from Section 2 is used on all experimental treatment arms separately, leading to test statistics ${\tilde{T}}_{k}$ , k=1,…,K . Using $\max({\tilde{T}}_{k}{)}_{k\in K}$ as the test statistic for $H_{K}$ , $H_{K}$ is rejected if $\max({\tilde{T}}_{k}{)}_{k\in K}\geq \Phi^{-1}(1-\frac{\alpha }{|K|})$ . This is the Bonferroni-Holm method in the simulations of Section 3.

In contrast, a ‘Dunnett-like’ closed test (see Magirr et al.^8^) is not straightforward. The marginal null distribution ${\tilde{T}}_{k}$ is N(0,1) , but the conditional correlation of ${\tilde{T}}_{k_{1}}$ and ${\tilde{T}}_{k_{2}}$ is not independent of the sample size modifications induced by the RAR procedure.

## 3. Simulation studies

To investigate the operating characteristics of the suggested design, we use the set-up of a trial with B=3 blocks (not including the burn-in), with block sizes (40, 40, 40) for all of the experimental treatments and (20, 20, 20) for the control. In the burn-in period, five patients are allocated to each of the treatments including the control. We set the true control mean $\mu_{0}=0$ , and α=0.05 . The auxiliary designs in all scenarios were simply random draws from a discrete uniform distribution on {1,…,K} , where K is the number of experimental treatment arms. The R code to generate the data and reproduce the simulation results can be found at https://github.com/dsrobertson/FWER_block_RAR.

### 3.1. Bayesian adaptive randomization

We compare the methods under a Bayesian adaptive randomization (BAR) scheme. Following Robertson and Wason,^4^ we use a block-randomized BAR scheme by Wason et al.^9^ The randomization probabilities $\left(\pi_{1},\ldots ,\pi_{K}\right)$ for the experimental treatments at the (j+1) th stage are given by $\pi_{k}=\frac{P(\mu_{k}>\mu_{0}|X=x{)}^{\gamma }}{\sum_{l=1}^{K}P(\mu_{l}>\mu_{0}|X=x{)}^{\gamma }}$ , where $P(\mu_{k}>\mu_{0}|X)$ is the posterior probability that $\mu_{k}$ is greater than $\mu_{0}$ given the observed data x , see the Supplemental material for full details. In our simulations, we set γ=0.5 . As well, since our proposal requires there to be at least one patient allocated to each experimental treatment per block, we ensure this is the case by allocating the last $K^{*}\geq 0$ patients in each block to each of the $K^{*}$ experimental treatments that have zero observations.

### 3.2. Error inflator scheme

To assess the FWER and power in a situation where type I control is known to be violated, we also investigate the allocation scheme presented in Section 2.3 of Robertson and Wason,^4^ adapted to block randomization. This rule keeps on allocating patients to treatment 1 (apart from one patient per block to each of the other experimental treatments) as long as the mean response of treatment 1 remains below a fixed threshold of 0.5. As soon as the fixed threshold is crossed, all subsequent patients not randomized to control are allocated with equal probability to the other experimental treatments (except for one patient per block on treatment 1 ). Full details are in the Supplemental material.

### 3.3. Examples of weights

Tables 1 and 2 show the weights from two simulations under BAR and the error inflator scheme, respectively. Throughout, we set $\mu_{1}=0,\mu_{2}=1$ for the experimental treatments. The proposal from Robertson and Wason,^4^ denoted RW, is compared with that from Section 2.

**Table 1.** Example test statistics and weights for BAR scheme with $\mu_{1}=0$ and $\mu_{2}=1$ . The standard ‘weights’ (i.e. realized sample sizes per arm) would be 65, 27 and 103 for the control, treatment 1 and treatment 2, respectively.

**Experimental treatment 1**
RW test statistic	−0.92
New test statistic	−1.11
	Block 1	Block 2	Block 3
RW experimental weights	45.87	41.53	23.20
New experimental weights	48.46	44.97	28.86
RW control weights	71.75	76.71	155.04
New control weights	42.95	42.95	42.95
**Experimental treatment 2**
RW test statistic	5.50
New test statistic	5.04
	Block 1	Block 2	Block 3
RW experimental weights	85.02	92.62	124.61
New experimental weights	80.14	84.57	101.30
RW control weights	61.93	60.85	57.94
New control weights	77.31	77.31	77.31

BAR: Bayesian adaptive randomization; RW: Robertson and Wason.

**Table 2.** Example test statistics and weights for the error inflator scheme with $\mu_{1}=0,\mu_{2}=1$ . The standard ‘weights’ (i.e. realized sample sizes per arms) would be 65, 122 and 8 for the control, treatment 1 and treatment 2, respectively.

**Experimental treatment 1**
RW test statistic	−1.87
New test statistic	−1.79
	Block 1	Block 2	Block 3
RW experimental weights	76.48	108.29	211.48
New experimental weights	69.00	86.05	130.34
RW control weights	59.90	56.32	53.57
New control weights	91.25	91.25	91.25
**Experimental treatment 2**
RW test statistic	0.76
New test statistic	2.92
	Block 1	Block 2	Block 3
RW experimental weights	53.67	35.71	8.16
New experimental weights	59.01	43.58	9.09
RW control weights	72.21	98.47	−62.83
New control weights	14.26	14.26	14.26

RW: Robertson and Wason.

In both examples, the weights can be very different for the two methods and also differ from both the observed sample sizes and the sample sizes in the auxiliary design. Both weight calculations up-weight the treatment arm with fewer allocations, while the RW weights tend to be more variable overall. The weight calculation from Robertson and Wason also produces negative weights for the control group for the error inflator scheme – something that is not possible with the calculation from Section 2. Note that the statements refer to a single simulation run. For the error inflator scheme, this is a case where treatment 2 crossed the threshold after block 0. Observed sample sizes and weights are very different for other simulation runs where this does not happen.

### 3.4. Simulation results

To investigate the performance of the various approaches, we conducted simulations for both the BAR and the error inflation scheme. As a standard comparison, we also provide simulation results for fixed (equal) randomization in the Supplemental material. The weighing approach from Robertson and Wason,^4^ the proposal from Section 2 and the naive approach (treating observed sample sizes as if they had been fixed in advance) were used. In all these approaches, the closed test procedure and the Bonferroni-Holm procedure are applied to adjust for the multiplicity arising from the testing of experimental treatments against a common control. In Tables 3 and 4, *disjunctive power* is the probability to reject at least one false null hypothesis (if there is one) and *error* is the FWER. Nominal test levels are assumed to be 5% .

**Table 3.** Familywise error rate and disjunctive power for BAR using block randomization with a fixed control allocation. There were $10^{5}$ simulated trials for each set of parameter values.

		Closed z -test		RW closed test		New closed test		z -test (Holm)		RW test (Holm)		New test (Holm)	
	Parameter values	Error	Power	Error	Power	Error	Power	Error	Power	Error	Power	Error	Power
1.	$\mu_{1}=\mu_{2}=0$	4.5	–	4.5	–	4.5	–	4.3	–	4.4	–	4.4	–
2.	$\mu_{1}=0$ , $\mu_{2}=0.5$	4.8	61.7	4.9	61.7	5.0	61.7	4.8	83.9	4.9	83.8	4.9	83.9
3.	$\mu_{1}=\mu_{2}=0.5$	–	94.6	–	94.6	–	94.6	–	92.3	–	92.4	-	92.4
4.	$\mu_{1}=\mu_{2}=\mu_{3}=0$	3.7	–	3.8	–	3.8	–	4.2	–	4.5	–	4.5	–
5.	$\mu_{1}=\mu_{2}=0$ , $\mu_{3}=0.5$	4.5	35.7	4.5	35.7	4.5	35.7	4.4	71.4	4.6	71.4	4.5	71.3
6.	$\mu_{1}=0$ , $\mu_{2}=\mu_{3}=0.5$	4.9	66.7	5.0	67.1	5.0	67.0	4.5	85.3	4.7	85.4	4.7	85.3
7.	$\mu_{1}=0$ , $\mu_{2}=0.25$ , $\mu_{3}=0.5$	4.5	50.6	4.6	50.8	4.6	50.7	3.6	72.3	3.7	72.5	3.7	72.4
8.	$\mu_{1}=\mu_{2}=\mu_{3}=0.5$	–	93.2	–	93.3	–	93.3	–	90.3	–	90.6	–	90.5

BAR: Bayesian adaptive randomization; RW: Robertson and Wason.

**Table 4.** Familywise error rate and disjunctive power for the error inflator scheme using block randomization with a fixed control allocation. There were $10^{5}$ simulated trials for each set of parameter values.

		Closed z -test		RW closed test		New closed test		z -test (Holm)		RW test (Holm)		New test (Holm)	
	Parameter values	Error	Power	Error	Power	Error	Power	Error	Power	Error	Power	Error	Power
1.	$\mu_{1}=\mu_{2}=0$	4.8	–	4.0	–	4.3	–	**6.3**	–	4.8	–	5.0	–
2.	$\mu_{1}=0$ , $\mu_{2}=1$	**7.3**	26.8	5.0	24.0	4.9	25.5	**7.2**	81.0	4.1	44.8	4.3	57.6
3.	$\mu_{1}=\mu_{2}=0.5$	–	94.7	–	93.0	–	93.8	–	92.0	–	88.4	–	90.0
4.	$\mu_{1}=\mu_{2}=\mu_{3}=0$	4.0	–	3.2	–	3.5	–	**5.8**	–	4.7	–	4.9	–
5.	$\mu_{1}=\mu_{2}=0$ , $\mu_{3}=1$	4.7	19.4	3.8	16.4	4.0	18.1	**6.1**	76.1	4.3	46.2	4.5	56.4
6.	$\mu_{1}=0$ , $\mu_{2}=\mu_{3}=1$	**7.3**	25.2	4.9	25.9	4.9	26.5	**7.0**	91.9	3.8	61.4	4.1	75.2
7.	$\mu_{1}=0$ , $\mu_{2}=0.5$ , $\mu_{3}=1$	**7.2**	23.0	4.7	22.0	4.8	23.2	**6.1**	80.1	3.3	50.0	3.6	61.4
8.	$\mu_{1}=\mu_{2}=\mu_{3}=0.5$	–	93.8	–	91.6	–	92.7	–	89.8	–	84.5	–	86.6

Note: Bold values indicate where the FWER is inflated above the nominal 5% level.

RW: Robertson and Wason.

Table 3 shows the results for the BAR scheme. As is well known, the closed test procedure has a slight power advantage if the treatments are equally effective, but is inferior when one of the treatments is effective, but the other(s) is not. FWER inflation did not occur in the simulations, even if the naive approach is used. The naive, the RW and the proposed approach lead to practically identical type I errors and power here. Although our new method does not perform better in terms of power, we consider it reassuring that it does not lose any power compared to the standard analysis either. Hence, in this specific case, there is no ‘price to pay’ for the guarantee of type I error control.

The results for the error inflator scheme are shown in Table 4. We see that the error inflator scheme indeed does not control the FWER. The inflation remains modest with a FWER not exceeding 7.5% in any of the simulation scenarios. This is, however, an inflation which is clearly beyond random simulation variation and which would not be acceptable in a confirmatory clinical trial. As expected, no FWER inflation arises when the two adaptive test methods are used. In line with what Robertson and Wason^4^ observed, there is a price to pay for the FWER control: both methods tend to suffer from power losses relative to their naive counterparts. The proposed procedure, however, has higher power than the RW approach and for some scenarios, the gain is substantial. For example, in scenarios 2, 5, 6 and 7, the power gain for the new Holm test compared with the RW test is between 10% and 20% in absolute terms. We hypothesize that this has to do with the fact that the variation of the weights is limited and cannot diverge as wildly from the observed sample sizes as they might with the RW approach (as illustrated in Table 2).

## 4. Case study

In this section, we revisit the case study used in Robertson and Wason^4^ of a phase II placebo-controlled trial in primary hypercholesterolemia to compare the effects of using the SAR236553 antibody with high-dose or low-dose atorvastatin, as compared with high-dose atorvastatin alone (Roth et al.^10^). The primary outcome was the least-squares mean percent reduction from baseline of low-density lipoprotein cholesterol. Patients were randomly assigned, in a 1:1:1 ratio, to receive 80 mg of atorvastatin plus placebo, 10 mg of atorvastatin plus SAR236553 or 80 mg of atorvastatin plus SAR236553. For convenience, we label these interventions as the ‘control’, ‘low dose’ and ‘high dose’, respectively.

We use the observed values from the trial and assume that the distribution of the primary outcome variable is distributed according to N(17.3/3.5,1) for the control, N(66.2/3.5,1) for the low dose and N(72.3/3.5,1) for the high dose, respectively. We suppose the trial is conducted using the BAR scheme presented in Section 3.1, with three blocks each of size 15 for the experimental doses (low and high doses). In the burn-in period, eight patients are allocated to low dose and eight patients to the high dose. Like in the actual trial, a total of 31 patients are allocated to the control, and 61 to the experimental doses.

Table 5 gives the results for a simulated trial with standard weights (i.e. realized sample sizes) 31 for the control, 27 for the low dose and 34 for the high dose. For the experimental doses, the realized breakdown per block (excluding the burn-in period) was (6,6,7) for the low dose and (9,9,8) for the high dose, respectively. We see that both the RW approach and our proposed approach give similar test statistics and weights in this case. In particular, given that all the observed p -values are < 0.001, using either approach would lead to rejecting the null hypothesis of no treatment effect.

**Table 5.** Test statistics, p -values and weights for a simulated block randomized trial using a BAR scheme. L = Low dose, H = High dose, C = Control. The standard weights (i.e. realized sample sizes) are 31 for the control, 27 for the low dose and 34 for the high dose.

	Low dose	High dose
z -test statistic	13.72 ( p<0.001 )	15.92 ( p<0.001 )
RW test statistic	14.00 ( p<0.001 )	15.58 ( p<0.001 )
New test statistic	14.25 ( p<0.001 )	15.24 ( p<0.001 )
RW weights	L=(26.06,26.06,29.21)	H=(36.01,36.01,30.44)
	C=(30.58,30.58,29.51)	C=(31.25,31.25,33.18)
New weights	L=(25.72,25.72,27.79)	H=(36.36,36.36,32.52)
	C=32.19	C=29.68

RW: Robertson and Wason.

## 5. Discussion

In this article, we have proposed an improved testing strategy based on the one by Robertson and Wason,^4^ which guarantees FWER control in the context of block-randomized response-adaptive trials with a fixed control allocation. Our proposal is simpler but is more restrictive as it is not applicable to fully sequential RAR or having an adaptive control allocation. However, our proposal guarantees that the weights are non-negative, and there can be substantial power gains in some settings.

As noted by Robertson and Wason,^4^ since the proposed testing procedure is based on the CIV principle, it has the additional important flexibility of being valid when the allocation is changed due to external information. Our proposal is also designed for normally distributed outcomes, although it can apply to other types of outcomes asymptotically. However, a natural extension of this work would be to work directly with binary endpoints (for example) and potentially apply the CIV principle to this setting.

The use of the CIV principle to ‘reweight’ the test statistics raises interesting questions around the design of optimal response-adaptive trials (i.e. the formulation of RAR procedures that optimize certain criteria). For example, some RAR procedures incorporate a formal power constraint, but this is based on standard test statistics. If an alternative testing strategy such as our proposed one is used, then there is a mismatch between the optimality criterion and the subsequent analysis of the trial.

More generally, it is important to remember that there can be trade-offs between the different objectives in a trial. For example, we have seen that insisting on the use of RAR methods which guarantee FWER control can lead to a substantial loss in power. As another example, more ‘extreme’ RAR procedures (i.e. those that skew the randomization probabilities close to 0 or 1) that perform well in terms of patient benefit metrics may conversely have low power and mean that analysis methods developed for a completely random sample are no longer appropriate. Hence the question of whether to use RAR as opposed to a fixed randomisation scheme is not a simple one, and crucially depends on the trial context and goals.

## Supplemental Material

sj-pdf-1-smm-10.1177_09622802231167437 - Supplemental material for Familywise error rate control for block response-adaptive randomization Supplemental material, sj-pdf-1-smm-10.1177_09622802231167437 for Familywise error rate control for block response-adaptive randomization by Ekkehard Glimm and David S Robertson in Statistical Methods in Medical Research
