# Supplementary material for: Familywise error rate control for block response-adaptive randomization
Source: Stat Methods Med Res. 2023 Apr 6;32(6):1193–202. doi: 10.1177/09622802231167437 (PMC10394397; doi:10.1177/09622802231167437)
Supplement: sj-pdf-1-smm-10.1177_09622802231167437 - Supplemental material for Familywise error rate control for block response-adaptive randomization [file sj-pdf-1-smm-10.1177_09622802231167437.pdf]

Supplemental material for "Familywise error rate control for block response-adaptive randomization" by Ekkehard Glimm and David S. Robertson

Web Appendix A Details of RAR procedures

Web Appendix A.1 Bayesian Adaptive Randomization (BAR)

Recall that the efficacy outcome for the ith treatment follows a $N(\mu_i, 1)$ distribution. We assign independent normal priors to the μ_i $(i = 0, 1, \ldots, K)$, such that $\mu_i \sim N(\mu_{i,0}, \sigma^2_{i,0})$. Let D_j denote the total number of patients allocated to the experimental treatments by the end of the jth block, and $\tilde{n}_{i,j}$ denote the number of patients allocated to treatment i by the end of the jth block. At stage $(j+1)$, when the outcomes $\boldsymbol{x} = (x_1, \ldots, x_{D_j})$ have been observed, the posterior for μ_i is as follows:

$$\mu_i \mid \boldsymbol{X} = \boldsymbol{x} \sim N\left(\frac{\sigma^2_{i,0}}{1+\tilde{n}_{i,j}\sigma^2_{i,0}} \sum_{k=1}^{D_j} \mathbb{1}_{\{a_k=i\}} x_k + \frac{\tilde{n}_{i,j}}{1+\tilde{n}_{i,j}\sigma^2_{i,0}} \mu_{i,0}\ ,\ \frac{\sigma^2_{i,0}}{1+\tilde{n}_{i,j}\sigma^2_{i,0}}\right).$$

In our simulations, for simplicity we set the priors $\mu_{i,0} = 0$ and $\sigma^2_{i,0} = 1$, while $\gamma = 0.5$. For our case study, we set the priors $\mu_{i,0} = 5$.

Web Appendix A.2 Error inflator scheme

Using the same notation as above, the allocation probabilities for block $j \in \{1, \ldots, J-1\}$, patient $k = D_j + 1, \ldots, D_{j+1}$ and treatment $l \in \{2, \ldots, K\}$ are:

$$P(a_k = 1) = \begin{cases} 0 & \text{if} \quad \sum_{i=1}^{D_j} \mathbb{1}_{\{a_i=1\}} \frac{X_i}{\tilde{n}_{1,j}} > 0.5 \\ 1 & \text{otherwise} \end{cases}$$

$$P(a_k = l) = \begin{cases} 1/K & \text{if} \quad \sum_{i=1}^{D_j} \mathbb{1}_{\{a_i=1\}} \frac{X_i}{\tilde{n}_{1,j}} > 0.5 \\ 0 & \text{otherwise} \end{cases}$$

Web Appendix B Fixed randomisation simulation study

Table 1 shows the familywise error rate and disjunctive power for fixed (equal) randomization.

	Closed z-test		RW closed test		New closed test		z-test (Holm)		RW test (Holm)		New test (Holm)	
Parameter values	Error	Power	Error	Power	Error	Power	Error	Power	Error	Power	Error	Power
1. $\delta_1 = \delta_2 = 0$	4.7	-	4.6	-	4.6	-	4.6	-	4.6	-	4.5	-
2. $\delta_1 = 0$, $\delta_2 = 0.5$	5.0	50.0	5.0	50.0	5.0	50.0	5.0	81.5	5.0	81.4	4.9	81.2
3. $\delta_1 = \delta_2 = 0.5$	-	94.6	-	94.6	-	94.5	-	92.3	-	92.2	-	92.1
4. $\delta_1 = \delta_2 = \delta_3 = 0$	3.7	-	3.7	-	3.7	-	4.6	-	4.6	-	4.6	-
5. $\delta_1 = \delta_2 = 0$, $\delta_3 = 0.5$	4.5	27.6	4.5	27.5	4.5	27.5	4.6	67.1	4.6	66.9	4.6	66.7
6. $\delta_1 = 0$, $\delta_2 = \delta_3 = 0.5$	5.0	55.7	5.0	55.7	5.0	55.7	4.7	83.6	4.7	83.5	4.7	83.4
7. $\delta_1 = 0$, $\delta_2 = 0.25$, $\delta_3 = 0.5$	4.6	42.6	4.6	42.5	4.6	42.5	3.8	69.8	3.8	69.7	3.8	69.5
8. $\delta_1 = \delta_2 = \delta_3 = 0.5$	-	93.2	-	93.1	-	93.1	-	90.1	-	90.0	-	90.0

Table 1: Familywise error rate and disjunctive power for fixed (equal) randomization. There were 10^5 simulated trials for each set of parameter values.
